# Gluteus medius contracture: A case report and review of the literature

**DOI:** 10.1097/MD.0000000000041311

**Published:** 2025-01-24

**Authors:** Yang Ma, Jiahao Gao, Fuwen Zheng, Jinshuo Tang, Chenyu Wang, Fengshuo Guo, Tong Liu, Jianlin Zuo

**Affiliations:** a Department of Orthopedics, China-Japan Union Hospital of Jilin University, Changchun, Jilin Province, PR China.

**Keywords:** arthroscopic surgery, gluteus medius contracture, Ober sign, open release

## Abstract

**Rationale:**

Bilateral gluteus medius contractures in adults are rare in clinical practice, with only a few cases reported. These contractures may result from repeated intramuscular injections during childhood. Understanding the clinical manifestations, diagnostic process, treatment, and outcomes can provide insights into effective management strategies.

**Patient concerns:**

A 28-year-old female presented with an abnormal walking posture characterized by an out-toeing gait. She reported long-standing difficulties in walking and sought medical attention to improve her mobility and quality of life.

**Diagnoses:**

The patient was diagnosed with bilateral gluteus medius contractures.

**Interventions:**

The patient underwent open surgical release of the contractures on both sides, followed by a structured rehabilitation program to restore muscle function and improve gait. Postoperative exercise guidance was provided to ensure optimal recovery.

**Outcomes:**

At the 1-year follow-up, the patient demonstrated significant improvement in gait, functional abilities, and overall quality of life. She expressed high satisfaction with the surgical and rehabilitative outcomes.

**Lessons:**

Once diagnosed with gluteus medius contracture, early surgical intervention is recommended. Through case reports and literature review, we have summarized the etiology, diagnostic methods, clinical characteristics, and existing treatment options for this condition.

## 1. Introduction

Gluteal muscle contracture is a clinical syndrome characterized by the degeneration and contracture of the gluteal muscle, iliotibial band, and fascia fibers. It is induced by various factors, resulting in limited hip joint movement, and characterized by typical gait and signs.^[1–3]^ This disorder was first described by Valderrama in 1970. According to the classification by Zhao et al,^[4]^ the most common contracture is gluteus maximus contracture. Bilateral gluteus medius contractures, especially in adults, are not commonly encountered in clinical practice.

## 2. Case presentation

**Figure 1.**
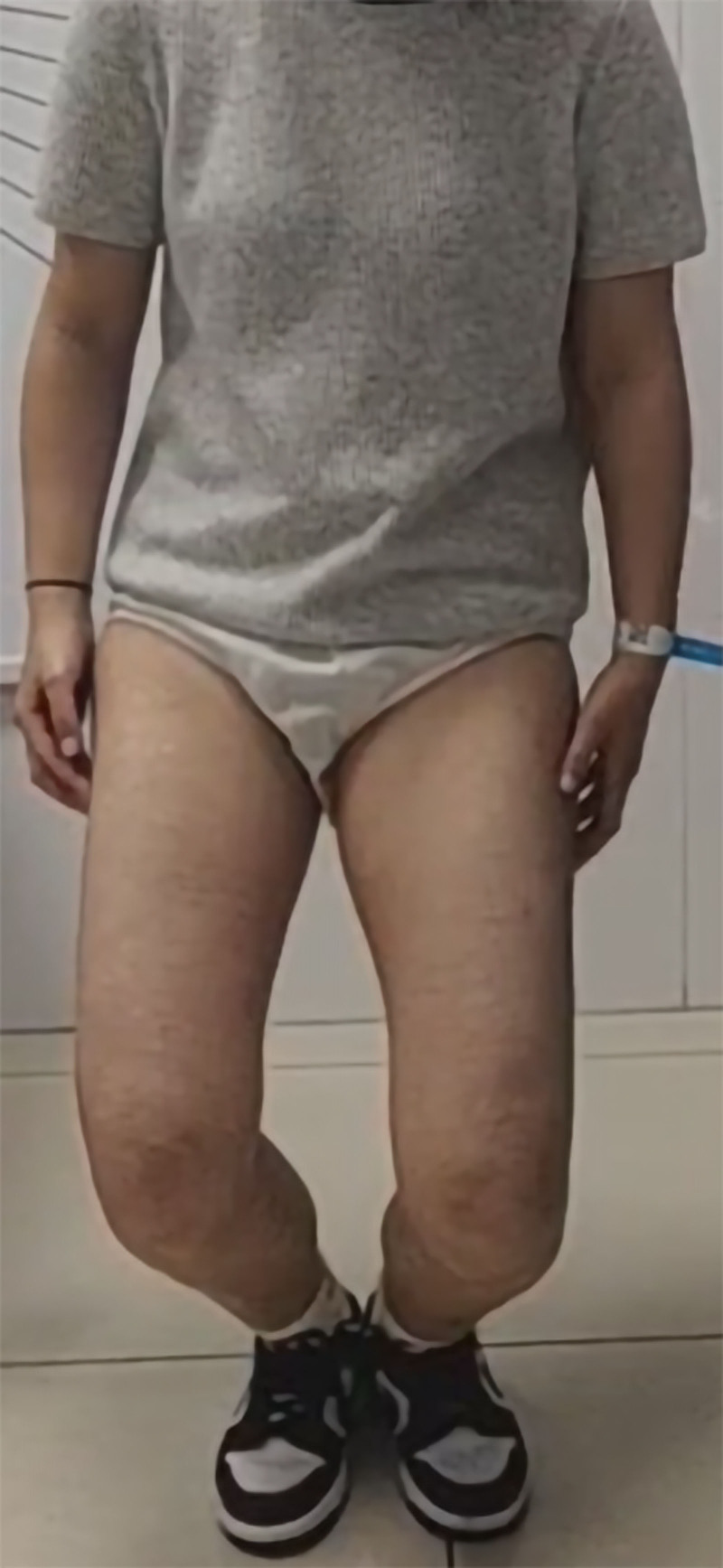
Preoperative clinical photograph of patient showing typical “frog position” on attempted squatting.

This study was informed that written consent was obtained from the patient by the Declaration of Helsinki (see Supplemental Digital Content 1, http://links.lww.com/MD/O291). A 28-year-old female presented to the orthopedic outpatient department with an abnormal walking posture and out-toeing gait,^[1]^ with both hips in the abduction and external rotation positions. She was unable to squat with her knees together, which forced her to assume a “frog position” when doing so (Fig. 1), and was unable to cross her legs. There was no history of trauma, relevant family medical history, or other congenital abnormalities, and she had not previously undergone any inpatient treatment. Physical examination revealed severe limitation of bilateral hip flexion and adduction, unequal length of the lower limbs, which was more severe on the left than on the right, and a positive Ober sign on both sides.

### 2.1. Imaging findings

**Figure 2.**
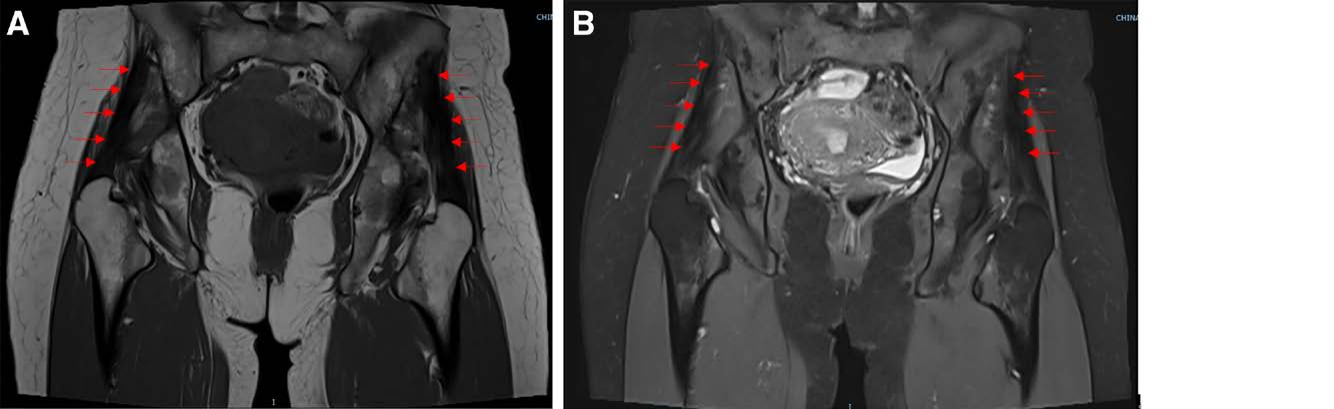
MRI reveals T1-weighted (A) and T2-weighted (B) images, showing significant atrophy of the bilateral gluteus medius, with a low-intensity band within this muscle on both T1- and T2-weighted images (indicated by arrows). These muscular changes are more prominent in the left thigh than in the right. MRI = magnetic resonance imaging.

Magnetic resonance imaging (MRI) revealed a significant reduction in the volume of the bilateral gluteus medius muscles, with a thickness decrease exceeding 50%. On T1-weighted images, the muscle tissue was extensively replaced by high-signal fat, rendering the gluteus medius markedly thinner, with a “cord-like” appearance, irregular contours, and retraction, indicative of severe muscle atrophy. The boundaries between the gluteus medius and the surrounding fascia were indistinct. On T2-weighted images, fibrosis was observed as extensive low-signal regions with distribution. The normal muscle texture was completely lost, replaced by a cord-like appearance with twisted and intertwined muscle fibers, and disorganized fiber bundle orientation. These findings indicate severe fibrosis. Additionally, the surrounding fascia was significantly thickened, forming tight adhesions with the gluteus medius (Fig. 2). Superficial Color Doppler Ultrasound showed significant volume reduction and thinning of the bilateral gluteus medius muscles, with nearly complete loss of normal muscle morphology. The muscle contours were blurred and irregular, and the boundary between the gluteus medius and surrounding fascia was indistinct, indicating severe atrophy and structural changes. The muscle exhibited uneven echogenicity, with enhanced low-echogenic bands representing fibrotic tissue, and the structure became more compact. Muscle fibers were arranged in a cord-like pattern, with the presence of strong echogenic foci and acoustic shadows. The normal muscle fiber structure was lost, replaced by fibrotic tissue and fat, resulting in widespread low-echogenic areas, suggesting severe fibrosis.

There were no obvious abnormalities in the tensor fascia lata or gluteus maximus. After being diagnosed with bilateral gluteus medius contracture in the outpatient department, the patient first attended the rehabilitation department of the authors’ hospital for conservative treatment, including massage, physiotherapy, short-wave diathermy, and active and passive stretching exercises; however, the results were unsatisfactory. Due to significant limitations in bilateral hip joint movement that severely affected the patient’s quality of life, surgical treatment was chosen after a thorough discussion of all treatment options.

**Figure 3.**
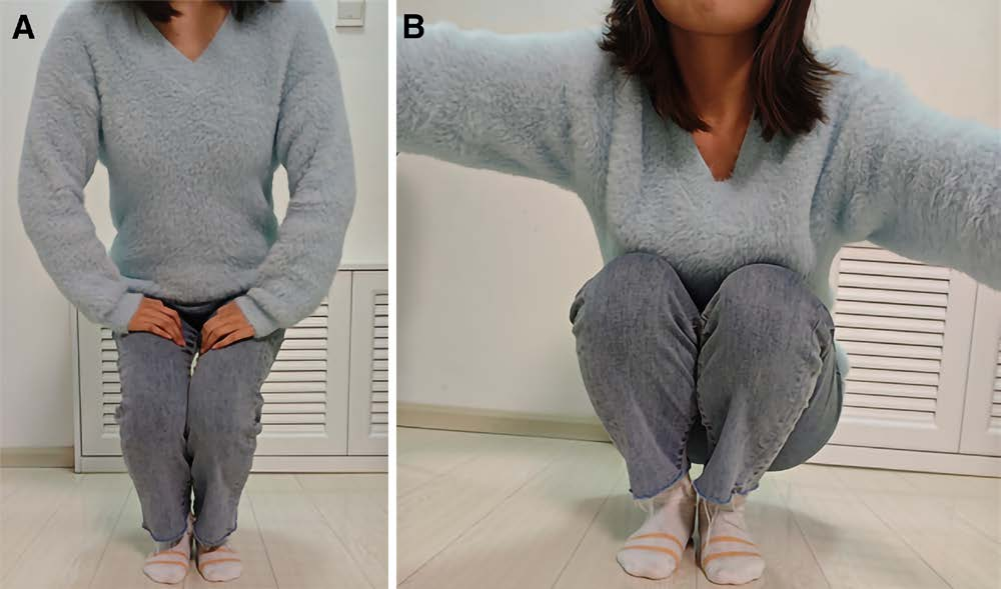
(A, B) Follow-up photographs showed that she could squat with her knees together.

**Figure 4.**
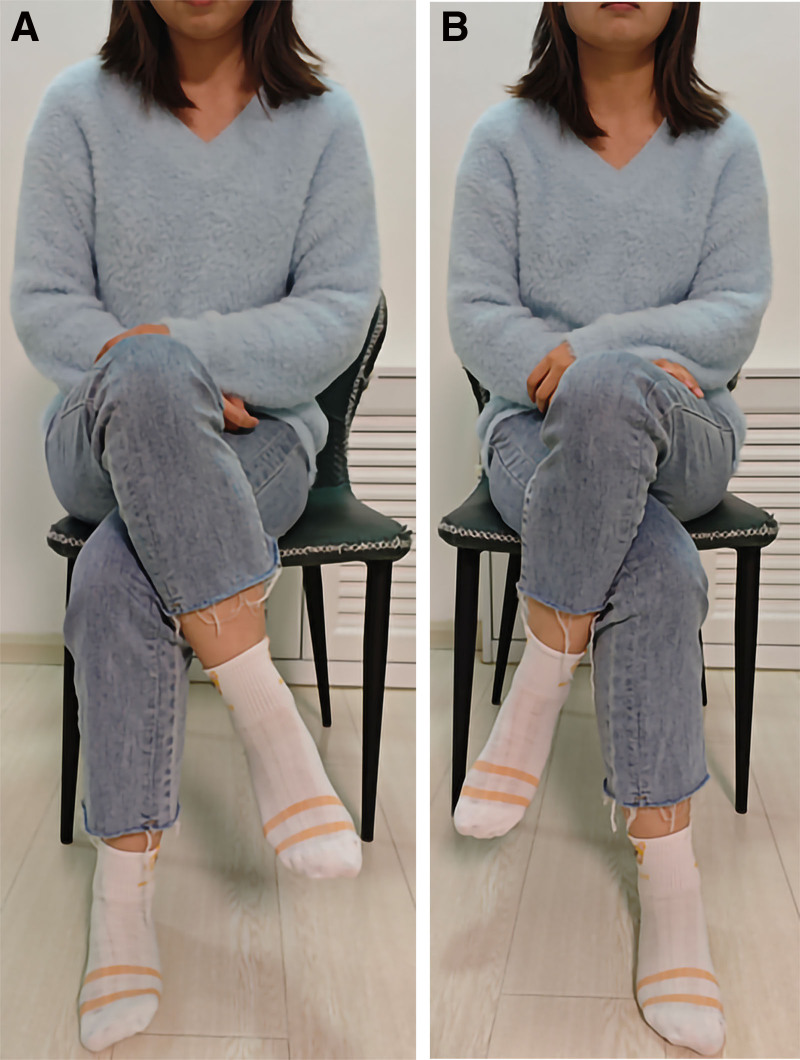
(A, B) Follow-up photographs showed that she could cross her legs.

Open surgery was performed bilaterally with the patient placed in the lateral decubitus position. After the completion of one side of the operation, the position was changed to the other side, and the bilateral operation was completed. A longitudinal incision was made at the center of the greater trochanter, and the subcutaneous tissue and fascia were cut longitudinally. During the operation, the gluteus medius muscle and gluteus fascia covering it exhibited obvious fibrous degeneration and contracture. The severity of the gluteus medius muscle on both sides differed, and the contracture on the left side was more severe than that on the right. Three-fourths of the contracted gluteus medius muscle and gluteal fascia were severed. The hip joint was restored during both extension and adduction. The Ober sign was negative, and the internal rotation of the hip was normal. Drainage was performed postoperatively to prevent hematoma formation, and the patient was guided to perform functional exercises after the drainage was removed on postoperative day 2. The exercise started with passive and active flexion of the knee and hip joints, and with the help of the physician, the patient was then asked to walk with a cross-gait and perform exercises such as squatting and crossing the legs in a sitting position. The patient was followed up for 1 year and showed improvement in all preoperative signs and symptoms. There was significant improvement in the limitation of bilateral hip joint range of motion with hip joint adduction exceeding 20°. The patient’s walking gait returned to normal and she was able to squat with her knees together (Fig. 3). Crossing of the legs was possible (Fig. 4), and the bilateral Ober sign was negative. No complications occurred and the patient was satisfied with the surgical results.

## 3. Literature review

### 3.1. Methods

We conducted a comprehensive search of the following biomedical databases: Embase, PubMed, and Web of Science, to identify relevant literature on gluteus medius contracture. We reviewed records published from the inception of each database through March 2024. with search fields restricted to titles and abstracts. The English search terms included “contracture,” “hip contracture,” “hip injuries,” “fibrosis,” “injections, adverse effects,” “injections, subcutaneous,” “injections, intramuscular,” and “gluteus medius,” “buttock,” “gluteal,” “gluteal region,” “hip,” with its synonyms included. Additionally, we manually searched the reference lists of all retrieved studies to ensure that all potentially eligible studies were included. The inclusion criteria were: 1. Document type: case reports, case series, and clinical studies with comprehensive case descriptions. 2. The patient was diagnosed with gluteus contracture, specifically involving the gluteus medius. 3. Include the essential components of a case report, including a patient history, comprehensive physical examination findings, diagnostic methods (such as laboratory tests and imaging studies), treatment process, and prognosis. Exclusion criteria were: 1. Content not related to gluteal contracture (excluding gluteus medius contracture). 2. The literature lacks critical information regarding diagnostic criteria, treatment outcomes, and prognosis. 3. Duplicate literatures and those of low quality, such as those with obvious errors (e.g., data fabrication or logical inconsistencies). 4. Article not in English.

### 3.2. Results

**Figure 5.**
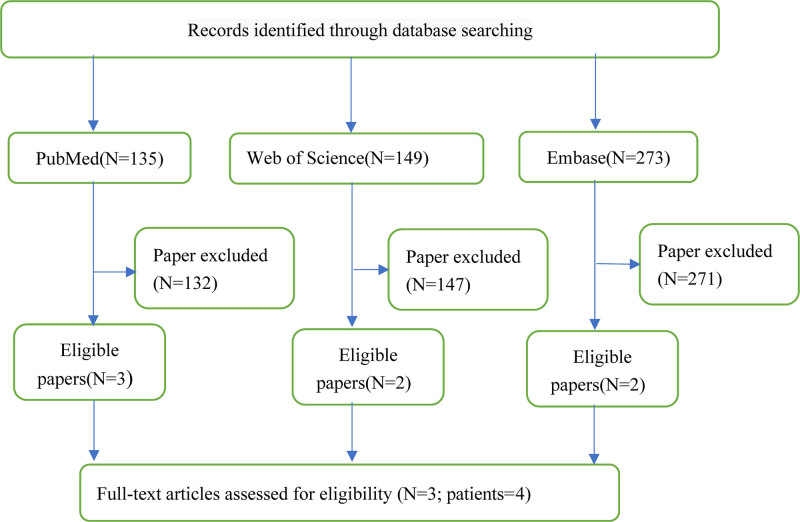
A flow diagram was presented for the retrieval and screening of the article.

Using the above search terms, We conducted searches in PubMed, Web of Science, and Embase, retrieving 135, 149, and 273 articles, respectively. After excluding studies that did not focus on gluteus medius contracture, lacked key information on diagnostic criteria, treatment outcomes, or prognosis, and non-English articles, we identified 3 studies in PubMed, 2 in Web of Science, and 2 in Embase. After removing duplicates, 3 articles remained. The literature search process is shown in Figure 5. To ensure consistency and standardization in data extraction, we created a unified table listing all required information categories, including “age at diagnosis, years,” “gender,” “laterality,” “probable reason,” “physical examination,” “treatment,” “outcome,” “follow-up, months.” This table was used to extract and summarize data from the 3 selected studies (Table 1). The patients ranged in age from 5 to 17 years (mean age: 12.25 years) and included 2 males and 2 females. Among them, 3 patients had bilateral gluteus medius and one had right gluteus medius contracture, and one had right gluteus medius and partial gluteus minimus contracture.

**Table 1:** Clinical characteristics of retrieved cases.

Author, year	Age at diagnosis, years	Sex	Laterality	Probable reason	Physical examination	Treatment	Outcome	Follow-up, months
Sirinelli et al, 1990^[7]^	11	Male	Right gluteus medius and gluteus minimus	History of buttock injections	1. Abduction and external rotation with limited flexion and adduction of the affected hip. 2. Dimpling of skin in the buttock area. 3. Apparent leg-length discrepancy.	Open operation	The patient recovered well without complications.	36
Jyoti et al, 2011^[10]^	5	Female	Bilateral gluteus maximus and gluteus medius	Idiopathic	1. Abduction and external rotation with limited flexion and adduction of the affected hip. 2. Unable to squat.	Operation	The patient recovered well without complications.	NA
You et al, 2015^[12]^	16	Female	Bilateral gluteus maximus and gluteus medius	History of buttock injections	1. Adduction and internal rotation dysfunction of hip. 2. Abnormal gait with out-toe walking. 3. Dimpling of skin around the gluteal region.	Arthroscopic surgery	The patient gained a satisfactory result without complications, snappings, and dysfunctions of the hip.	3
	17	Male			1. Adduction and internal rotation dysfunction of the hip. 2. Abnormal gait with out-toe walking. 3. Ober sign.	Open surgery was performed first, followed by arthroscopic surgery.	The effect of open surgery was poor, and then arthroscopic surgery was performed. The patient gained a satisfactory result without complications, snappings, and dysfunctions of the hip.	3

NA = not available.

### 3.3. Etiology and diagnosis

According to the medical histories of the reviewed cases, 3 patients had bilateral gluteus medius and part of the gluteus maximus contracture, and 1 had right gluteus medius and part of the gluteus minimus contracture. These patients were diagnosed on the basis of their clinical manifestations and physical examinations. All patients manifested limited hip joint movement, especially hip adduction and internal rotation dysfunction, including abnormal gait, walking outside the toes, inability to squat or sit with 2 legs on both knees and a positive Ober sign.^[5]^ Specifically, 3 of the 4 patients elicited a popping sound during hip rotation, and 1 had a leg-length discrepancy. In the upright position, the legs were unequal, the pelvis was significantly tilted, and the right leg was significantly shortened. However, pelvic radiography revealed no abnormalities in the right hip joint. One patient presented with a snapping hip on clinical examination. In the current literature, most cases of gluteus medius contracture are secondary to repeated intramuscular injections. In these cases, we reviewed 3 patients with a history of injections in the gluteal region. The relationship between injections and muscle atrophy is plausible; as such, it is reasonable to speculate that gluteus injections may be the cause.

### 3.4. Treatment

Addressing gluteus medius contractures involves a range of interventions, including nonsurgical approaches, various surgical treatments, programmed rehabilitation, and physical therapy. However, establishing a consensus on standard treatments remains challenging. Three patients refused to undergo surgical treatment due to the absence of overt pain or severe movement limitations coupled with concerns about potential surgical risks. Among the remaining patients, 3 chose to undergo surgical intervention, which was performed unilaterally or bilaterally, depending on the severity of symptoms and expected functional improvement.

## 4. Discussion

Researchers have explored diverse hypotheses regarding the etiology of gluteus medius contractures, considering factors such as congenital, traumatic, and genetic influences.^[1,6,7]^ Repeated intramuscular injections constitute the most extensively documented cases,^[7]^ whereas idiopathic occurrences are exceptionally rare. In a case-control study, Chung and Ko^[8]^ reported a statistically significant correlation between intramuscular injections and gluteus fiber contractures. These 2 case-control studies examined the likelihood of a history of injections in patients with gluteal contracture compared with controls. These findings revealed a higher prevalence of gluteal muscle injections among patients with gluteal contractures and a positive correlation between the frequency of injections and the development of muscle fibrosis.^[1,8,9]^ Jakkani et al^[10]^ documented a case of idiopathic gluteus medius contractures. The patient, devoid of a history of repeated intramuscular injections, trauma, or similar symptoms in family members, had a potentially congenital origin. Zhao et al^[4]^ reported the presence of congenital gluteal contractures in both parents and children, confirming the possibility of a congenital etiology; however, the pathogenesis and genetic mechanisms remain unclear.

The diagnosis of gluteus medius contracture is based on patient history, signs, and imaging findings. In the case described, the patient’s physical presentation was contingent on the severity of the disease, with restricted hip movement being a characteristic feature. This may manifest as limitations in buckling, an inability to perform adduction, and positive outcomes in the active buckling experiment with crossed legs, along with a positive Ober sign.^[5,11]^ In this case, bilateral gluteus medius contracture was definitively diagnosed based on MRI, ultrasonography (USG), and the patient’s clinical presentation. Imaging studies revealed severe muscle atrophy and fibrosis, which provided direct radiological evidence for the diagnosis. A review of the literature demonstrates that different studies have adopted distinctive diagnostic approaches for gluteus medius contracture: Sirinelli et al^[7]^ utilized clinical findings in combination with CT and X-ray imaging to accurately localize the lesion and confirm the diagnosis; Jakkani et al^[10]^ identified characteristic features of muscle atrophy and fat replacement using MRI, such as linear streaks of hyperintensity on T1-weighted images, which further supported the diagnosis. Similarly, You et al^[12]^ highlighted the value of three-dimensional reconstructed CT by analyzing clinical manifestations and detecting significant underdevelopment of the gluteus medius muscle, thereby establishing the diagnosis. MRI is frequently used to evaluate the extent of muscle involvement, including fibrosis and muscle atrophy.^[13]^ Chen et al^[13]^ identified intramuscular fiber cords and muscle atrophy as primary MRI features of fibromuscular contractures. The fiber cords exhibit a low-signal intensity across all MRI sequences, with the most pronounced visibility on fat-suppressed images. Furthermore, computed tomography may reveal gluteal muscle atrophy, calcification, necrosis at the injection site, frizzled fascial bands, and widening of the gluteal space. USG is characterized by thinning of the involved gluteal muscles and the presence of hyperechoic bands within the muscle bundles, suggesting fibrosis.^[1,14]^ Clinical presentation is the cornerstone for diagnosing gluteus medius contracture; however, imaging examinations play a crucial role not only in confirming the diagnosis but also in assessing the severity of the condition and ruling out other diseases.^[1,13,14]^ The features observed in this case are consistent with those reported in the literature, underscoring the complementary value of imaging modalities. Notably, the marked muscle atrophy and fibrosis identified in this patient further validate the diagnostic sensitivity of MRI and USG while providing valuable insights for optimizing diagnostic workflows. Compared with previous reports, the more pronounced imaging characteristics in this case offer critical clues for disease staging and severity assessment.

Currently, treatment options for the gluteus medius muscle include both nonsurgical and surgical approaches. Nonsurgical treatment is proposed for mild cases and as an adjunct to surgical treatment and mainly includes massage, physical therapy, short-wave diathermy, and active and passive stretching,^[4]^ although few studies have confirmed the efficacy of nonsurgical treatment in patients with gluteal contractures. It has been argued that once contracture is established, nonoperative treatment has no role. Zhao^[4]^ reported that among 49 patients, nonsurgical treatment was effective in only 38%, despite a very strict rehabilitation protocol. The patients in Zhao’s study exhibited varying degrees of contracture, lacked a strictly defined control group, and had an insufficient sample size, making it difficult to draw definitive conclusions regarding the ineffectiveness of nonsurgical treatment.^[4]^ Currently, studies on nonsurgical treatment for gluteus medius contracture are extremely limited, with a lack of high-quality evidence, such as prospective or controlled trials, to provide universal and reliable efficacy assessments. This field still presents significant research gaps that require further exploration. The patient we report chose surgical intervention after nonsurgical treatments failed. In contrast, the 4 patients in our review opted for surgery without attempting nonsurgical treatments. Surgical treatment is the gold standard method of treatment for all established cases of gluteal muscle contracture.^[15–17]^ These include traditional open release and endoscopic release, each of which has advantages and disadvantages. Liu et al^[18]^ did not advise surgery in children <5 years of age because they were unable to adhere to a strict postoperative rehabilitation regimen.

The open surgical release has been performed for decades with excellent outcomes. It is indicated in all established cases but is highly recommended in severe cases because a wide incision provides appropriate exposure, enabling the division of fibrotic bands under direct vision. It involves a variable length and shape of the skin incision, usually in the lateral position over the buttock and greater trochanter. There are a variety of incision options, including small, straight, and S-shape incisions. In this case, a longitudinal incision approximately 8 cm in length centered on the tip of the greater trochanter was made. The incision was aesthetically pleasing and could fully expose the main release part and expose the gluteus medius, gluteus minimus, piriformis, and posterior joint capsule to ensure a smooth operation. Simultaneously, the operation was performed around the greater trochanter with few complications. A small incision is usually not completely released and prone to complications; therefore, it is not recommended. An S-shape incision can lead to large trauma and an unaesthetic incision. In contrast, a longitudinal incision centered on the tip of the greater trochanter is the first choice. Zhao^[4]^ performed 187 open surgical releases and obtained 97% good-to-excellent results. Postoperatively, 62 patients developed hypertrophic scarring, 6 experienced acute hematoma formation, 3 had wound infections, and 1 suffered a wound dehiscence. Al Bayati et al^[2]^ reported 7 cases in Iraq in which conventional open release was performed. The patients were followed up for 2 to 12 months, and the results were excellent in all cases, without any known complications. These well-known complications of traditional open surgery negatively impact patients’ functional and aesthetic satisfaction, particularly among adolescents. As a result, arthroscopic release has been proposed as an alternative.^[19]^ You et al^[12]^ reported 4 cases, one of which involved a patient who underwent open surgery at the age of 13. The initial outcome was unsatisfactory, and after 4 years, the patient underwent arthroscopic release, achieving a satisfactory result without any complications. Endoscopic release of gluteal muscle contracture is an emerging technique, and only a limited number of studies have been performed, assuming that arthroscopic release of the contracted muscle would avoid extensive surgical trauma caused by the incision. However, in a study by Fu et al,^[19]^ there was no statistically significant difference between endoscopic release and conventional open surgical release in terms of operative duration, complications, clinical outcome, or 1-year recurrence rate. Unfortunately, 4 patients with severe contracture in the endoscopic group experienced unsatisfactory results after arthroscopic treatment and were treated with open surgery. This suggests that endoscopic technology has specific limitations. Although arthroscopic surgery is superior to open surgery in terms of cosmetic satisfaction and trauma, it requires the surgeon to have a wealth of knowledge about instrumentation, procedures, and anatomical layers. At the same time, an arthroscope may not be as effective in visualizing deeper structures such as the gluteus medius, gluteus minimus, piriformis muscle, and joint capsule, and the large amount of normal saline used to create an operative field may negatively affect healthy muscles.^[12,17]^ Ye^[16]^ proposed a new minimally invasive open-release technique, reporting excellent outcomes based on their evaluation criteria. However, to date, no other studies have utilized this technique. The authors did not adequately describe the technical difficulties and limitations associated with it. Although the technique is simple and easy to perform, with few complications, it relies on the patient’s anatomical landmarks for localization. These landmarks can vary significantly across different age groups and heights. In the report, 3 patients experienced an acute rupture of a branch of the circumflex femoral artery at the femoral neck during surgery, resulting in patient dissatisfaction. A major concern is that this procedure is performed blindly through small incisions, which increases the risk of incomplete release and potential neurovascular injuries. In the review of 4 reported cases, all were managed through surgical intervention, including 2 open surgeries, 1 arthroscopic surgery, and 1 case where arthroscopic surgery was performed following the failure of an initial open surgery. All cases achieved satisfactory outcomes. In our reported case, cutting 3/4 of the contracted gluteus medius and fascia resulted in favorable outcomes. Sirinelli et al^[7]^ achieved satisfactory results by completely severing the gluteus medius attached to the ilium; however, this approach often led to Trendelenburg gait,^[20]^ indicating that complete resection is inappropriate and should be avoided even in cases of severe contracture. The surgical details in other reported cases were not adequately described.

Postoperative rehabilitation is crucial for rapid recovery and optimal clinical outcomes.^[18]^ This can be avoided by wound drainage, as in the present case. We recommend that patients perform functional exercises after the drain is removed.^[12]^ The exercise starts with passive and active flexion of the knee and hip. We recommend placing the legs on a continuous passive motion machine and continuously increasing the range of motion of the hip and knee. Engaging in daily activities, such as walking on a line, straightening the waist, and adducting the hip joint multiple times is beneficial. Perform squats with the knees together, ensuring that the heel remains on the ground, to gradually achieve hip and knee flexion beyond 90°. Sitting with the legs crossed and straightening the waist to bring the back of one thigh into contact with the front of the other thigh extends the hip muscles and promotes hip joint movement. In addition, instructing the leg in adduction, abduction, intorsion, and extorsion enhances muscle flexibility. It is essential to gradually increase the intensity of these exercises to optimize outcomes. Collectively, these measures aimed to restore hip muscle strength and improve range of motion.

A review of the literature reveals that current research on gluteus medius contracture primarily consists of case reports or single-center studies. While these reports hold significant clinical value, they have notable limitations, focusing mainly on diagnosis and presentation, with insufficient in-depth analysis of individual cases and detailed surgical descriptions. Systematic studies are scarce, and only Zhao et al^[4]^ have proposed a classification for pediatric gluteus medius contracture, which lacks clear and standardized criteria, limiting its generalizability. Comparisons of the efficacy of different surgical approaches have not been fully explored. Larger-scale prospective studies, controlled trials, and multicenter collaborative research are needed to validate the long-term outcomes of various treatments. Furthermore, the molecular mechanisms underlying gluteus medius contracture should be investigated to provide a foundation for targeted therapies.

## 5. Conclusion

Gluteus medius contractures are relatively rare. In the current study, we review the etiology, diagnosis, and treatment strategies for gluteus medius contracture and present a case of bilateral gluteus medius contracture in an adult female. Our patient, although presenting late, experienced good outcomes based on the literature and our experience. This finding suggests that even late interventions can yield good outcomes and should be offered to patients. We believe that a thorough history and clinical examination are essential for the diagnosis of gluteus medius contracture, and that prompt diagnosis and surgical treatment can significantly improve quality of life.

## Author contributions

**Conceptualization:** Tong Liu, Jianlin Zuo.

**Funding acquisition:** Jianlin Zuo.

**Investigation:** Yang Ma, Jiahao Gao, Fuwen Zheng, Jinshuo Tang, Chenyu Wang, Fengshuo Guo.

**Methodology:** Fengshuo Guo.

**Resources:** Jianlin Zuo.

**Visualization:** Yang Ma, Jiahao Gao, Jianlin Zuo.

**Writing – original draft:** Yang Ma.

**Writing – review & editing:** Jinshuo Tang, Chenyu Wang, Tong Liu, Jianlin Zuo.

## Supplementary Material


